# Clinical Outcomes of Trimethoprim/Sulfamethoxazole in Critically Ill Patients with *Stenotrophomonas maltophilia* Bacteremia and Pneumonia Utilizing Renal Replacement Therapies

**DOI:** 10.3390/jcm13082275

**Published:** 2024-04-14

**Authors:** Wasim S. El Nekidy, Khaled Al Zaman, Emna Abidi, Diaa Alrahmany, Islam M. Ghazi, Rania El Lababidi, Mohamad Mooty, Fadi Hijazi, Muriel Ghosn, Mohamed Askalany, Mohamed Helal, Ahmed Taha, Khaled Ismail, Jihad Mallat

**Affiliations:** 1 Department of Pharmacy Services, Cleveland Clinic Abu Dhabi, Abu Dhabi P.O. Box 112412, United Arab Emirates; khaledalzaman@gmail.com (K.A.Z.); abidiee@clevelandclinicabudhabi.ae (E.A.); ellabar@clevelandclinicabudhabi.ae (R.E.L.); 2 Cleveland Clinic Lerner College of Medicine, Case Western Reserve University, Cleveland, OH 44106, USA; 3 Pharmaceutical Care Department, Directorate General of Medical Supplies, Ministry of Health, Muscat 3110, Oman; diaa.alrahmany@yahoo.com; 4 Arnold and Marie Schwartz College of Pharmacy, Long Island University, Brooklyn, NY 11201, USA; islam.ghazi@liu.edu; 5 Department of Infectious Disease, Cleveland Clinic Abu Dhabi, Abu Dhabi P.O. Box 112412, United Arab Emirates; mootym@clevelandclinicabudhabi.ae; 6 Department of Nephrology, Cleveland Clinic Abu Dhabi, Abu Dhabi P.O. Box 112412, United Arab Emirates; hijazif@clevelandclinicabudhabi.ae (F.H.); ghosnm@clevelandclinicabudhabi.ae (M.G.); 7 Critical Care Institute, Cleveland Clinic Abu Dhabi, Abu Dhabi P.O. Box 112412, United Arab Emirates; askalam@clevelandclinicabudhabi.ae (M.A.); helalm@clevelandclinicabudhabi.ae (M.H.); tahaa2@clevelandclinicabudhabi.ae (A.T.); ismailk@clevelandclinicabudhabi.ae (K.I.)

**Keywords:** trimethoprim/sulfamethoxazole, hemodialysis, continuous renal replacement therapy (CRRT), bacteremia, pneumonia, *Stenotrophomonas maltophelia*

## Abstract

**Background**: The clinical outcomes of usual doses of Trimethoprim–sulfamethoxazole (TMP/SMZ) for treating *S. maltophilia* in critically ill patients on renal replacement therapies (RRT) have not been established. We sought to assess the clinical outcomes of TMP/SMZ in patients with sepsis utilizing RRT. **Methods**: A retrospective study was performed on all critically ill adult patients with *S. maltophilia* infections who received RRT between May 2015 and January 2022. The primary endpoint was clinical cure while the secondary endpoints were microbiologic cure, 30-day infection recurrence, and mortality. **Results**: Forty-five subjects met the inclusion criteria. The median age was 70.0 [interquartile range (IQR): 63.5–77] years, 57.8% were males, and the median body mass index was 25.7 [IQR: 22–30.2] kg/m^2^. Clinical success and failure were reported in 18 (40%) and 27 (60%) cases, respectively. There was no significant difference between the 30-day reinfection rates of both groups; however, mortality was significantly higher in the clinical failure group, involving 12 patients (44.4%), versus none in the clinical success group (*p* = 0.001). The median daily dose of TMP/SMZ upon continuous veno-venous hemofiltration was 1064 [IQR: 776–1380] mg in the clinical cure group vs. 768 [IQR:540–1200] mg in the clinical failure group (*p* = 0.035). Meanwhile, the median dose for those who received intermittent hemodialysis was 500 [IQR: 320–928] mg in the clinical success group compared to 640 [IQR: 360–1005] mg in the clinical failure group (*p* = 0.372). A total of 55% experienced thrombocytopenia, 42% hyperkalemia, and 2.2% neutropenia. The multivariable logistic regression analysis showed that the total daily dose at therapy initiation was the only independent factor associated with clinical success after adjusting for different variables including the body mass index [Odds ratio 1.004; 95% confidence interval: (1–1.007), *p* = 0.044]. **Conclusions**: Although the *S. maltophilia* isolates were reported as susceptible, TMP/SMZ with conventional doses to treat bacteremia and pneumonia in critically ill patients utilizing RRT was associated with high rates of clinical and microbiologic failure as well as with mortality. Larger outcomes and pharmacokinetics studies are needed to confirm our findings.

## 1. Background

*Stenotrophomonas maltophilia* (*S. maltophilia)* is a ubiquitous, Gram-negative, multidrug-resistant bacteria increasingly recognized as a cause of hospital-acquired infections (HAIs). The rising prevalence of *S. maltophilia* catheter-related bacteremia, peritonitis, and pneumonia in patients on renal replacement therapy is causing significant morbidity and mortality among this population [1,2,3,4]. Indwelling devices, chronic respiratory disease, an immunocompromised state, prolonged antibiotic use (mainly of carbapenems), and long-term hospitalization or intensive care unit (ICU) admission are all significant risk factors for *S. maltophilia* infections [3,4]. Sulfamethoxazole in combination with trimethoprim, Trimethoprim–sulfamethoxazole (TMP/SMZ), is an antibiotic that inhibits dihydrofolate reductase and is considered the drug of choice for the treatment of *S. maltophilia* infections. A plethora of studies have proven the effectiveness of TMP/SMZ as a monotherapy or in combination in the treatment of pneumonia or bacteremia caused by *S. maltophilia* [5,6,7]. TMP/SMX’s high tissue penetration, long half-life, bioequivalence of oral and intravenous dosage forms, and broad-spectrum antimicrobial activity against Gram-positive bacteria including community-acquired Methicillin-resistant *Staphylococcus aureus* (MRSA), Gram-negative bacteria, fungi, and some protozoal species make it an optimal antimicrobial therapy in a slew of infections including urinary tract infections, prostatitis, pneumonia, and bacteremia [8,9,10,11]. TMP/SMX is used to treat *S. maltophilia* pneumonia and bacteremia in adults at a dose of 15–20 mg/kg every 6 to 8 h. No dose adjustment is needed in patients with estimated creatinine clearance (eCrCl) > 30 mL/min while one-half of the usual dosage is recommended when eCrCl is 15 to 30 mL/min; lower doses (25 to 50%) are prescribed if eCrCl is less than 15 mL/min [12,13,14]. In patients on continuous renal replacement therapy (CRRT), both oral and intravenous TMP/SMX are substantially removed through CRRT [15,16], and for that, no dosage adjustment is necessary [16,17]. On the other hand, in patients on intermittent hemodialysis (IHD), dose recommendations for patients with eCrCl <15 mL/min and not on dialysis should be followed; doses due on dialysis days should be administered after hemodialysis [18]. The pharmacokinetics of the same antibiotic combination indicate half-times of 9 to 11 and 10 to 15 h for sulfamethoxazole and trimethoprim, respectively, in individuals with normal kidney function. End-stage kidney disease patients present with longer intervals of 20 to 50 h and 24 h [19,20]. Moreover, pharmacokinetic studies have also demonstrated the necessity of reduced dosage in patients with creatinine clearance <30 mL/min [20]. Although scarce data exist about drug usage in patients on different renal replacement therapy (RRT) modalities, it has also been demonstrated that hemodialysis sessions could lead to significant reductions in the usage of both antibiotics to half-time to reach normal values [20,21]. Therefore, TMP/SMZ dosage in this cohort of patients remains difficult and understudied [9].

The protocol of TMP/SMZ dosing at our institution for the treatment of pneumonia and bacteremia caused by *S. maltophilia* adults suggests a dose of 15 to 20 mg/kg/day of TMP divided every 6 to 8 h in patients with normal kidney function while it is recommended to use lower doses (25 to 50% less) in those with compromised kidney function [12,14]. However, the TMP and SMZ removal while on CRRT was documented to be substantial [15,16]; hence, our protocol suggests not to reduce the dose, especially in critically ill patients [16,17]. On the other hand, in patients utilizing IHD, the doses should be reduced like in those with eCrCl <15 mL/min, with the doses administered after dialysis-on-dialysis days and as scheduled on non-dialysis days.

However, there was a lack of clinical outcome data to confirm the efficacy and safety of these dosings in critically ill patients utilizing IHD and continuous veno-venous hemofiltration (CVVH). Hence, the purpose of this study was to investigate the clinical outcomes of the used doses of TMP/SMZ at our institution in patients utilizing renal replacement therapy.

## 2. Methods

### Study Design and Case Selection

After the receipt of the institution’s research ethics committee approval, adults (aged more than 17 years) with sepsis admitted to our institution between May 2015 and January 2022 were retrospectively included if they received RRT (either IHD or CVVH or switched between both depending on their hemodynamic stability), had confirmed bacteremia or pneumonia secondary to *S. maltophilia* bacteria, and were treated with TMP/SMZ during the same period of RRT.

Collected culture samples included sputum, bronchoalveolar lavage, and blood cultures. Demographics, baseline characteristics, microbiologic data, TMP/SMZ dose, frequency, and infection-related parameters were collected.

The primary outcome was the clinical success rate whereas the secondary outcomes were 30-day and 90-day mortality, the microbiological cure rate, the recurrent infections, and the development of adverse events consequent to antimicrobial therapy.

## 3. Patient Data

Gender, age, body mass index, underlying comorbidities, microbiological data (sample source, site of infection, susceptibility test, and received antimicrobial therapy), and symptoms of infections (to exclude colonization) such as fever and relevant clinical and laboratory findings (C-reactive protein, procalcitonin, and white blood cell count) were collected. Clinical information on ICU admission, each patient’s Acute Physiology and Chronic Health Evaluation (APACHE) VI score, vasopressors and invasive mechanical ventilation requirements, and the RRT modalities were collected. Repeated blood and sputum cultures were also collected.

### 3.1. Patient Consent Statement and Ethical Approval

The procedures used in this study adhered to the tenets of the Declaration of Helsinki. Informed consent was waived by the Cleveland Clinic Abu Dhabi Research Ethics Committee in view of the retrospective nature of the present study, and all the procedures performed were part of the patients’ routine care.

### 3.2. Definitions

The diagnosis was based on clinical and microbiologic criteria as documented by the treating physician. Clinical cure was defined as the resolution of infection signs and symptoms as reported in the treating physician’s notes. Clinical failure was defined as either the persistence of functional symptoms for more than 72 h after initiating the study drugs (TMP/SMZ) or documentation as a failure by the treating physician and requiring changing of antimicrobials or other therapies. Microbiologic cure was defined as no isolation of causative pathogen after repeating culturing from the same site at the end of the therapy. Efficacy parameters included both the clinical and microbiological clearance of the bacteria as well as inflammatory markers (white blood cells [WBCs], C-reactive protein, and procalcitonin). Recurrence was defined as repeated positive culturing of the same pathogen with clinical symptoms in the follow-up 30-day period after the resolution of the initial episode.

The microbiology results were interpreted according to the Clinical and Laboratory Standards Institute (CLSI). Hyperkalemia was defined as potassium level of >4.9 mmol/L, leukopenia was defined as WBC count < 4.0 cells/mm^3^, and thrombocytopenia was defined as platelet level of <50 cells/mm^3^.

End-stage kidney disease patients were defined as those having a creatinine clearance rate falling below 15 mL/min. Hospital-acquired infections were infections that occurred after 48 h of a patient’s hospital admission; remaining infection episodes were counted as community-acquired diseases. Indwelling devices were catheters used for various purposes such as vascular access or respiratory support. Immunocompromised individuals included those with human immunodeficiency virus (HIV)/acquired immunodeficiency syndrome (AIDS) and/or cancer, organ transplant recipients, and those undergoing immunosuppressive therapies (like chemotherapy or long-term corticosteroid use).

Pneumonia was defined based on the following factors: (1) a new shadow appearing on chest X-ray or computed tomography scan; (2) fever or hypothermia; and (3) signs and symptoms related to respiratory infection, including two or more of the following: cough, purulent sputum, abnormal auscultatory findings, signs of respiratory failure, signs of dyspnoea, and an increased amount of tracheal aspirate in mechanically ventilated patients [22].

### 3.3. Renal Replacement Therapies

At our institution, we perform both CVVH and IHD modalities. Critically ill patients could be switched between both modalities based on their hemodynamic stability. The CVVH hemofiltration replacement fluid rate is usually between 20 and 25 mL/kg/h with an average net ultrafiltration rate between 0 and 300 mL/h as tolerated and a median blood flow rate of 180 to 200 mL/min. The median time of patients on CVVH at our institution is 20 h per day (based on CVVH interruptions). Most patients in this study received replacement fluids at 70% prefilter and 30% post-filter. The commonly used filters were the Prismaflex^®^ M150 (Baxter Healthcare Corporation, Deerfield, IL, USA) and, to a lesser extent, the M100. IHD was performed for between 3.5 and 4 h per session and 3 to 4 times weekly. Blood flow rate ranged between 250 and 400 mL/min, dialysate flow rate between 500 and 600 mL/min, and ultrafiltration rate between 500 and 3500 mL per treatment session based on clinical situations. The most used filters in HD were the Polyflux^®^ 140 H and 170 H (Baxter Healthcare Corporation, Deerfield, IL, USA).

## 4. Statistical Analysis

Data are expressed as means ± standard deviations (SDs) or medians [interquartile range (IQR)] according to the distribution of variables. Proportions were used as descriptive statistics for categorical variables. The normality of data distribution was assessed using the Shapiro–Wilk test. Comparisons of values between independent groups were performed using the Mann–Whitney *U* test or two-tailed Student’s *t* test, as appropriate. Analysis of the discrete data was performed using *χ*^2^ test or Fisher’s exact test when the numbers were small.

A multivariable logistic regression analysis was used to identify significant independent factors that were associated with clinical success. Variables that were associated with clinical success (*p* < 0.1) in univariate analysis and those that might potentially influence the clinical success occurrence, such as the doses and durations of the antibiotics, concomitant use of antibiotics, and the severity of the inflammatory state, were entered into the model. The potential problem of colinearity was evaluated using the Spearman or Pearson correlation coefficient before running the analysis. Goodness-of-fit of the model was assessed using Hosmer–Lemeshow’s test.

A two-sided *p*-value < 0.05 was used as the criterion to determine statistical significance in this study. All statistical analyses were performed using SPSS statistical package version 28 for Windows (IBM, Armonk, NY, USA).

## 5. Results

Forty-five patients met the inclusion criteria and were categorized into two groups according to the clinical success or failure outcomes. The median age of the sample was 70.0 [IQR: 63.5–77.0] years. However, it showed no statistically significant difference between those in the clinical success group, where it was 67.5 years [IQR: 63.7–74.0], and those who failed the treatment, for whom it was 70.0 years [IQR: 63.0–79.0]. A total of 57.8% were males.

Parameters such as the APACHE IV score and prevalence of comorbidities like diabetes mellitus, coronary artery disease, hypertension, dyslipidemia, liver disease, cerebrovascular events, respiratory disease, and congestive heart failure did not show significant differences between the success and failure groups. Of note, weight was significantly higher in the clinical success group compared to the clinical failure group (80.0 ± 19.7 vs. 67.9 ± 19.1 kg, *p* = 0.029). There was also a trend of higher body mass index in the clinical success group compared to the clinical failure group (Table 1). The baseline characteristics and demographics of the study sample are presented in Table 1.

Treatment outcomes are presented in Table 2. Most patients were diagnosed with pneumonia (93%) vs. bacteremia (7%). Patients with end-stage kidney disease represented 44% of the sample, with 73.3% receiving CVVH and 26.7% receiving IHD, while 49% received both IHD and CVVH depending on their hemodynamic stability. No statistically significant differences were found between the clinical and failure groups regarding the RRT modalities (Table 2). Fourteen patients (77.8%) received norepinephrine in the clinical success group compared to twenty-five patients (92.6%) in the clinical failure group. This difference was not statistically significant (*p* = 0.20). Also, 16 patients (89%) received invasive mechanical ventilation in the clinical success group compared to 27 patients (100%) in the clinical failure group. This difference was not statistically significant (Table 2).

All *S. maltophilia* isolates were reported as susceptible to TMP/SMZ. However, the microbiologic cure rate was reported to be 13.3%, the 30-day recurrence rate was 15.5%, the 30-day overall mortality rate was 26.7%, and the 90-day mortality rate was 48.9% (Table 2). Of note, the microbiological cure rate was significantly higher in the clinical success group compared to the clinical failure group (22.2% vs. 7.4%, *p* = 0.001). Also, no patients died at the 90-day point in the clinical success group compared to 48.9% of the patients dying in the clinical failure group (*p* < 0.001). TMP/SMZ dosing was captured in 29 patients while utilizing CVVH and in 23 patients while utilizing IHD. The C-reactive protein concentration at the end of therapy, documented in 39 patients, was 40.6 mg/L [IQR:11.6–74.7] in the clinical success group compared to 78.70 mg/L [IQR: 50.65–187.30] in clinical failure patients (*p* = 0.015). However, no statistically significant differences were observed between the clinical and failure groups regarding procalcitonin and WBCs at antibiotic initiation (Table 2). Among the 45 patients, 55.5% experienced thrombocytopenia, 1% leukopenia, and 42% hyperkalemia. Only hyperkalemia was significantly different between both groups (*p* = 0.035).

Ninety-one percent of the study population received TMP-SMX initially via the intravenous administration route while the remaining received an oral formulation. However, 35.5% of the patients were switched from intravenous to oral administration during the treatment course, and 22.2% received concomitant antimicrobial therapy, which was not significantly different between both groups (*p* = 0.489). Overall, concomitant antibiotics were reported in ten patients: six patients were taking ceftazidime with TMP/SMZ, two patients were taking moxifloxacin with TMP/SMZ, and one patient was taking levofloxacin with TMP/SMZ. Two patients were taking levofloxacin and ceftazidime with TMP/SMZ. There were no significant differences in therapy duration between the clinical success and clinical failure groups after 10 [IQR: 4.75–20.25] days and 12 [IQR: 7–21] days (Table 3).

The total median daily dose of TMP/SMZ while on CVVH was significantly higher in the clinical success group compared to the clinically failed group (1064 [IQR: 776–1380] mg vs. 768 [IQR: 540–1200] mg, respectively; *p* = 0.035) (Table 3). The total daily dose of TMP/SMZ, body mass index, duration of antibiotics therapy, concomitant antibiotics, norepinephrine use, and C-reactive protein at the end of therapy were included into the multivariate logistic regression model. This analysis showed that the drug’s total daily dose is the only significant risk factor of clinical success [Odds ratio 1.004; 95% CI (1–1.007)] after adjustment for the different variables including the body mass index (Table 4).

## 6. Discussion

In this study, we reported the outcomes of the use of TMP/SMZ in the management of pneumonia and bacteremia secondary to *S. maltophilia* infections in critically ill patients utilizing RRT. Our results revealed a predominance of clinical failure with a rate of 60%. Moreover, a heightened incidence of microbiologic failure (86%) and increased all-cause 90-day mortality rates (49%) were observed despite the susceptibility of all *S. maltophilia* isolated to TMP/SMZ. Our data also suggest that combination therapies did not improve clinical success or microbiologic cure (*p* = 0.489) (Table 3). The multivariate logistic regression showed that only the total daily dose was the main risk factor of clinical success after adjusting for different variables including the body mass index (Table 4). To our knowledge, this is the largest study to investigate the clinical outcomes of TMP/SMZ in critically ill patients utilizing CVVH and/or IHD.

A plethora of studies have compared the efficacy of TMP/SMZ monotherapy in the management of *S. maltophilia* infections. Indeed, Wang et al. retrospectively compared the efficacy of levofloxacin (n = 63) vs. TMP/SMZ (n = 35) against *S. maltophilia* with more than 50% of the patients having pneumonia [16]. The clinical cure, microbiologic cure, and mortality were 55%, 62%, and 24%, respectively, with no significant differences between groups concluding the equal efficacy of both agents. The median daily dose in that study was 7.8 mg/kg/day. However, critically ill patients were excluded from that study and only 25% of the sample had chronic kidney disease in all stages including ESKD utilizing dialysis [23].

Similarly, Nys et al. retrospectively compared the efficacy of TMP/SMZ (n = 45) compared to levofloxacin (n = 31) against *S. maltophilia* with mainly pneumonia (92%). The clinical cure, microbiologic cure, and mortality rates were reported to be 79%, 82%, and 14%, respectively [24]. The study included only 10 patients treated with hemodialysis (12%). Also, 72% of the total population was admitted to the critical care unit at the time of the therapy initiation. The median dose of the drug was 10.3 mg/kg/day [24].

Furthermore, Cho et al. retrospectively compared the efficacy of TMP/SMZ (n = 51) vs. levofloxacin (n = 35) with only 11 (12.8%) patients utilizing CRRT and IHD and 22 (25%) in the critical care unit. The utilized dose was 15 to 20 mg/kg/day, adjusted to the kidney function as needed. The authors reported a 30-day mortality rate of 24.4%, and 23% experienced adverse events in the TMP/SMZ reported as cytopenia and hepatotoxicity [25].

Unlike in the aforementioned studies, we observed significantly higher rates of clinical failure, lower rates of microbiologic cure, and higher 30- and 90-day mortality rates. These findings could be explained by the fact that all our patients were critically ill and utilizing RRT. Furthermore, the median daily dose of TMP/SMZ used in our study was significantly higher than those used in previous studies (Table 3). Of note, the utilized dose for those who had clinical success while treated with CVVH was significantly higher than the dose for those who experienced clinical failure and also higher than the median doses used in patients with normal kidney function in the above-mentioned studies.

In another retrospective descriptive study, Guerci et al. described the outcomes of different antimicrobials in 282 critically ill and mechanically ventilated patients from 25 critical care units in France on *S. maltophilia*-associated pneumonia [26]. The survival analysis did not show benefit from combination therapy, nor from a prolonged duration of more than 7 days. Additionally, Shah et al., in a retrospective single-center cohort study that included 252 patients, compared the efficacy of combination therapy (n = 38) vs. monotherapy (n = 214) in the management of *S. maltophilia* pneumonia [27]. Most of these patients (76.2%) were admitted into the ICU, and 69.4% received invasive mechanical ventilation. They observed no significant difference between the combination therapy group and the monotherapy group regarding the 7-day clinical success (47.7% vs. 39.7%, respectively; *p* = 0.38). Also, after controlling for immunocompromised patients, polymicrobial pneumonia and APACHE II score were identified as confounding factors; there was no significant difference regarding the clinical response. Additionally, the microbiological cure rates were not significantly different between the combination therapy and monotherapy groups (42.9% vs. 59.5%, respectively; *p* = 0.44). Furthermore, after controlling for confounders using multivariate logistic regression analysis, they found no significant differences in microbiologic cure, recurrence, or 30-day infection-related mortality rates between the combination and monotherapy groups. However, all-cause mortality was significantly higher in the combination therapy group than in the monotherapy group (39.5% vs. 22.9%, *p* = 0.03) [27]. We found similar results in our critically ill patients utilizing RRT who received combination therapy. Nevertheless, according to the Infectious Disease Society (IDS) 2023 guidance on the treatment of antimicrobial resistant Gram-negative infections, the suggested general approach for the treatment of infections caused by *S. maltophilia* is a combination therapy (i.e., TMP-SMX, minocycline/tigecycline, cefiderocol, or levofloxacin), at least until clinical improvement is observed [28].

The overall 30-day and 90-day mortality rates (26.7% and 48.9%, respectively) observed in our study were high. This finding was consistent with the results of Shah et al. where the overall reported 30-day all-cause mortality rate was 25.4% [27]. Also, in the Guerci et al. study, the observed overall in-hospital mortality rate was 49.7% [26]. Interestingly, only SOFA score and age were independently associated with mortality, and no specific pneumonia- or antimicrobial-therapy-related factors impacted the outcome [26].

Relatively recent recommendations of optimal antimicrobial dosing during renal replacement therapy [29] have reported that both TMP and SMX are removed via continuous veno-venous hemodiafiltration (dialysate rate: 1.5 L/h, ultrafiltration rate: 1.5 or 2.55 L/h) to a significant degree [15]. Steady-state TMP peak concentrations between 5 and 10 mg/L and steady-state SMX peak concentrations between 100 and 200 mg/L were proven in an in vitro experimental setting after an initial dose of TMP 10 mg/kg/day, and the corresponding SMX dose (50 mg/kg/day) and that same dose were therefore associated with efficacy against *P. jirovecii* and were proven to be appropriate, when divided q12h, as parts of an initial dose to be considered in patients undergoing CVVH or continuous veno-venous hemodiafiltration (ultrafiltration/dialysate rates: 1, 2, 3, and 6 L/h) [16]. Another study comparing standard-dose (≥6 single-strength (SS) TMP-SMX 80 mg/400 mg tablets/week) and low-dose groups (<6 single-strength tablets/week) in HIV-uninfected adult patients who were undergoing hemodialysis and were administered TMP-SMX for Pneumocystis pneumonia prophylaxis confirmed the appropriateness of a low-dose TMP-SMX regimen for prophylaxis with a balanced profile of adverse events due to TMP-SMX administration [30].

There is a lack of consistent evidence for dosing TMP/SMZ in critically ill patients utilizing CVVH and/or IHD [31]. For the same reason, we were not able to compare our dosage in patients on HD or CRRT or on switching modalities with reported dosages from the cited studies. Thus, therapeutic drug monitoring is needed while dosing antimicrobials in patients utilizing RRT. The recent in vitro time-kill study demonstrated antimicrobial monotherapy’s failure to treat *S. maltophilia* infection. However, bactericidal activity was only achieved with combination therapies [32]. This in vitro study was contradicted by the clinical findings of both Guerci et al. and Shah et al.’s studies [26,27].

Our study presents the inherent limitations of a retrospective observational study. Also, it was a single-center study with a small sample size and time-limited experience. Thus, our data could not be generalized without previously extended research. However, to our knowledge, this was the largest study to investigate the clinical outcomes of TMP/SMZ in critically ill patients utilizing CVVH and/or IHD. Moreover, patients on concomitant antibiotic treatments were not excluded, which rendered treatment courses often heterogeneous; therefore, control for additional anti-infective treatment, other than that of a TMP/SMX monotherapy, was difficult. Consequently, clinical outcomes may have been influenced by other factors unrelated to *S. maltophilia* infection such as the presence of multiple comorbidities or the severity of the disease. Also, the levels of TMP/SMX were not measured in the serums of our patients. Furthermore, the isolation of *S. maltophilia* in patients’ airway cultures does not always mean infection and can reflect colonization with a low bacterial load [4,33,34,35]. However, as shown in Table 2, inflammatory markers were elevated, suggesting infection in our patients. Also, we did not collect the previous antimicrobial treatments received by the patients before the *S. maltophilia* infection, which was another limitation of the study. It has been shown that the pre-administrations of antipseudomonal β-lactam, anti-MRSA, and broad-spectrum β-lactam antibiotics are risk factors for *S. maltophilia* pneumonia and bacteremia [36,37,38,39,40]. This might have influenced the findings of this study. Also, we did not collect the main reasons for hospitalization before the development of hospital-acquired infections caused by *S. maltophilia*.

## 7. Conclusions

Our findings indicated that although the isolates were reported as susceptible, TMP/SMZ with conventional doses to treat bacteremia and pneumonia caused by *S. maltophilia* in critically ill patients utilizing RRT was associated with high rates of both clinical and microbiologic failure as well as mortality. Larger outcomes and pharmacokinetics studies are needed to confirm our findings.

## Figures and Tables

**Table 1:** Demographics and baseline characteristics of the study sample.

	All Patients (n = 45)	Clinical Success (n = 18)	Clinical Failure (n = 27)	*p*-Value
Males, n (%)	26 (57.8)	10 (55.5)	16 (59.2)	0.464
Age, year	70.0 [63.5–77.0]	67.5 [63.7–74.0]	70.0 [63.0–79.0]	0.594
Weight, kg	72.8 ± 20.0	80.0 ± 19.7	67.9 ± 19.1	0.029
Body Mass Index, kg/m^2^	25.7 [22.1–30.2]	26.9 [22.7–32.7]	24.1 [20.4–27.6]	0.067
APACHE IV score	93.1 ± 38.5	87.6 ± 27.7	95.8 ± 43.1	0.568
Diabetes Mellitus, n (%)	22 (48.9)	9 (50.0)	13 (48.1)	1
Coronary Artery Disease, n (%)	5 (11.1)	0 (0.0)	5 (18.5)	0.073
Hypertension, n (%)	24 (53.3)	12 (66.7)	12 (44.4)	0.223
Dyslipidemia, n (%)	12 (26.7)	4 (22.2)	8 (29.6)	0.735
Liver disease, n (%)	15 (33.3)	3 (16.7)	12 (44.4)	0.063
Cerebrovascular event, n (%)	14 (31.1)	4 (22.2)	10 (37.0)	0.343
Respiratory disease, n (%)	35 (77.8)	13 (72.2)	22 (81.4)	0.489
Congestive Heart Failure, n (%)	6 (13.3)	3 (16.7)	3 (11.1)	0.67

APACHE, acute physiology, and chronic health evaluation. Data are expressed as means ± SDs, medians [25–75 interquartile range], or counts (percentages).

**Table 2:** Treatment outcomes.

	All Patients (n = 45)	Clinical Success (n = 18)	Clinical Failure (n = 27)	*p*-Value
Acute Kidney Injury, n (%)	25 (55.5)	9 (50.0)	16 (59.2)	0.559
Acute Kidney Injury on CKD, n (%)	11 (23.9)	2 (11.1)	9 (32.1)	0.241
End Stage Kidney Disease, (n %)	20 (44.4)	9 (50.0)	11 (40.7)	0.559
CCVH, n (%)	33 (73.3)	11 (61.1)	22 (81.4)	0.175
Intermittent Hemodialysis, n (%)	12 (26.7)	7 (38.9)	5 (18.5)	0.175
Switched between RRT Modalities, n (%)	22 (48.9)	10 (55.6)	12 (44.4)	0.55
Pneumonia, n (%)	42 (93.3)	16(88.9)	26 (96.2)	0.555
Bacteremia, n (%)	3 (6.7)	2 (11.1)	1 (3.7)	0.555
Culture site				0.028
Sputum culture, n (%)	30 (66.7)	15 (83.3)	15 (55.5)	
Bronchoalveolar Lavage culture, n (%)	12 (26.7)	1 (5.6)	11 (40.7)	
Blood, n (%)	3 (6.7)	2 (11.1)	1 (3.7)	
Norepinephrine, n (%)	39 (86.7)	14 (77.8)	25 (92.6)	0.199
Mechanical ventilation, n (%)	43 (95.5)	16 (88.9)	27 (100)	0.155
The temperature at therapy initiation, °C	36.9 [36.5–37.4]	36.9 [36.4–37.4]	36.90 [36.5–37.5]	0.71
Fever, n (%)	4 (8.9)	1 (5.6)	3 (11.1)	0.64
WBC at therapy initiation, mm^3^	18.7 [14.6–29.3]	18.1 [14.0–20.7]	21.0 [15.5–33.6]	0.203
CRP at antibiotic initiation, mg/L	102.9 [62.3–206.4]	94.5 [65.3–210.8]	105.8 [55.8–192.4]	0.705
CRP at end of antibiotic, mg/L	67.1 [32.6–106.5]	40.6 [11.6–74.7]	78.7 [50.6–187.3]	0.015
Procalcitonin at antibiotic initiation, ng/L	2.1 [0.9–5.4]	2.2 [0.8–5.5]	2.1 [1.0–4.5]	0.863
TMP/SMZ susceptible, yes, n (%)	45 (100)	18 (100)	27 (100)	
Repeated culture, n (%)	28 (62.2)	11 (61.1)	17 (62.3)	0.979
Microbiologic cure, n %)	6 (13.3)	4 (22.2)	2 (7.4)	<0.001
30-day reinfection, n (%)	7 (15.5)	3 (16.7)	4 (14.8)	1
90-day reinfection, n (%)	14 (31.1)	6 (33.3)	8 (29.6)	0.748
30-day mortality, n (%)	12 (26.7)	0 (0.0)	12 (44.4)	0.001
90-day mortality, n (%)	22 (48.9)	0 (0.0)	22 (81.5)	<0.001
Adverse events, n (%)				
Low platelets	25 (55.5)	9 (50.0)	16 (59.3)	0.474
leukopenia	1 (2.2)	0 (0.0)	1 (3.7)	1
Hyperkalemia	19 (42.2)	4 (22.2)	15 (55.5)	0.035

CCVH: continuous veno-venous hemofiltration; RRT: renal replacement therapy; CRP: C-reactive protein; WBC: white blood cell count; TM/SMZ: Trimethoprim–sulfamethoxazole. Data are expressed as means ± SDs, medians [25–75 interquartile range], or counts (percentages). ‘Low platelets’ is defined as platelet level < 50,000/μL. Leukopenia is defined as WBC count < 4 × 10^9^/L. Hyperkalemia is defined as potassium level > 4.9 mEq/L.

**Table 3:** TMP/SMZ dosing in different RRT modalities.

	All Patients (n = 45)	Clinical Success (n = 18)	Clinical Failure (n = 27)	*p*-Value
The total daily dose in CVVH, mg	960 [620–1200]	1064 [776–1380]	768 [540–1200]	0.035
Total daily dose in CVVH, mg/kg	12.9 [9.7–14.9]	13.8 [9.8–15]	12.76 [9.7–14.6]	0.435
Total daily dose in IHD, mg	590 [332–97]	500 [320–928]	640 [360–1005]	0.372
Total daily dose in IHD, mg/kg	7.8 [5.0–14]	5.5 [4.7–11.7]	8 [5.0–15]	0.218
Therapy Duration, day	12 [6–21]	10 [4–20]	12 [7–21]	0.291
TMP/SMZ Intravenous administration, n (%)	41 (91.1)	18 (100)	23 (85.2)	0.138
TMP/SMZ administration switched from IV to oral, n (%)	16 (35.5)	7 (38.9)	9 (33.3)	0.758
Combination of antibiotics, n (%)	10 (22.2)	5 (27.8)	5 (18.5)	0.489

CCVH: continuous veno-venous hemofiltration; TM/SMZ: Trimethoprim–sulfamethoxazole; IV: intravenous; IHD: intermittent hemodialysis. Data are expressed as means ± SDs, medians [25–75 interquartile range], or counts (percentages).

**Table 4:** Multivariable logistic regression for clinical cure.

Variable	Odds Ratio (95% CI)	*p*-Value
Total daily dose of TMP/SMZ	1.004 (1–1.007)	0.044
Body mass index	1.04 (0.923–1.17)	0.523
Duration of antibiotics therapy	0.987 (0.92–1.05)	0.69
Concomitant antibiotics	1.2 (0.15–9.8)	0.864
Norepinephrine	0.2 (0.02–2.2)	0.186
C-Reactive Protein at the end of therapy	0.98 (0.958–1.005)	0.118

TMP/SMZ: Trimethoprim–sulfamethoxazole; CI: confidence interval.

## Data Availability

The data presented in this study are available on request from the corresponding author (for ethical reasons).
